# Multiple β Forms of Saturated Monoacid Triacylglycerol Crystals

**DOI:** 10.3390/molecules25215086

**Published:** 2020-11-02

**Authors:** Seiya Takeguchi, Arisa Sato, Hironori Hondoh, Mio Aoki, Hidetaka Uehara, Satoru Ueno

**Affiliations:** 1 The Nisshin OilliO Group, Ltd.1 Shinmori-cho, Isogo-ku, Yokohama 235-8558, Japan; s-takeguchi@nisshin-oillio.com (S.T.); h-uehara@nisshin-oillio.com (H.U.); 2 Graduate School of Integrated Sciences for Life, Hiroshima University, 1-4-4 Kagamiyama, Higashi-Hiroshima 739-8528, Japan; sachertorte310@gmail.com (A.S.); gchpnfam05@gmail.com (M.A.); sueno@hiroshima-u.ac.jp (S.U.); 3 School of Food and Nutritional Sciences, University of Shizuoka, 52-1 Yada, Suruga-ku, Shizuoka 422-8526, Japan

**Keywords:** triacylglycerol, polymorphism, DSC, synchrotron radiation X-ray diffraction

## Abstract

We have investigated the polymorphism of triacylglycerol (TAG) crystals as they affect the qualities such as shelf life, mouth feel, and texture of chocolate and other products. Saturated monoacid TAGs, like trilaurin, are considered as models for TAG crystallization; however, there is still debate about the number of their polymorphs that exist. In this study, we characterized a set of novel polymorphs, β forms of saturated monoacid TAGs, which were obtained via different pathways depending on the crystallization history, by polarized light microscopy, X-ray diffraction, and differential scanning calorimetry. Saturated monoacid TAGs were crystallized as the unstable polymorphs, the α or β’ forms first, and then they were transformed into β forms by solid–solid transformations. The β form that had transformed from β’ changed its morphology by a polymorphic transformation, while the β form made from the α form kept its spherulite morphology. The β forms obtained showed different melting points. Additional heat treatment promoted further polymorphic transformation. Four novel β forms were found for each of the saturated monoacid TAGs, trilaurin, trimyristin, tripalmitin, and tristearin. They showed similar polymorphism with the same subcell packing.

## 1. Introduction

Triacylglycerol (TAG), an ester of three fatty acids and glycerol, is the main constituent of fats and oils. TAG crystals are well known to show polymorphism and monotropic transformations depending on the crystallization temperature, cooling rate, and storage temperature [1]. Three typical polymorphs are well characterized based on the crystal packing of fatty acid moieties; β, β’, and α in the order of the melting point [2]. This packing is characterized by a “subcell”, which is defined as the type of cross-sectional packing of aliphatic chains. The zigzag planes of the hydrocarbon chains in each polymorph are either parallel (β), perpendicular (β’), or random (α) to each other [1].

Monotropic transformations in TAGs are well studied since they are significant for the application. Cocoa butter, the main ingredient of chocolate, shows complex polymorphism, having six polymorphs, I to VI, including two β’ and two β forms [3,4,5]. In commercial chocolate, TAG exists as a well-organized β_V_ form. These will transform into the β_VI_ form since that is the most stable polymorph of cocoa butter [3]. This transformation is the origin of the deterioration of chocolate products known as fat blooming [6,7]. The cocoa butter crystals grow larger by this polymorphic transformation, and the chocolate surface roughens. This rough surface scatters light and looks white. Hence, chocolate with fat bloom has a cloudy visual appearance that is not preferred and has a poor melting quality in the mouth because of the high melting point of the β_VI_ form of cocoa butter. The polymorphic transformation in margarine promotes a granular crystal formation [8], which results in a sandy texture and decreases the spreadability. Therefore, gaining an understanding of the polymorphism of TAG crystals is important to maintain good quality in these products.

**Figure 1:**
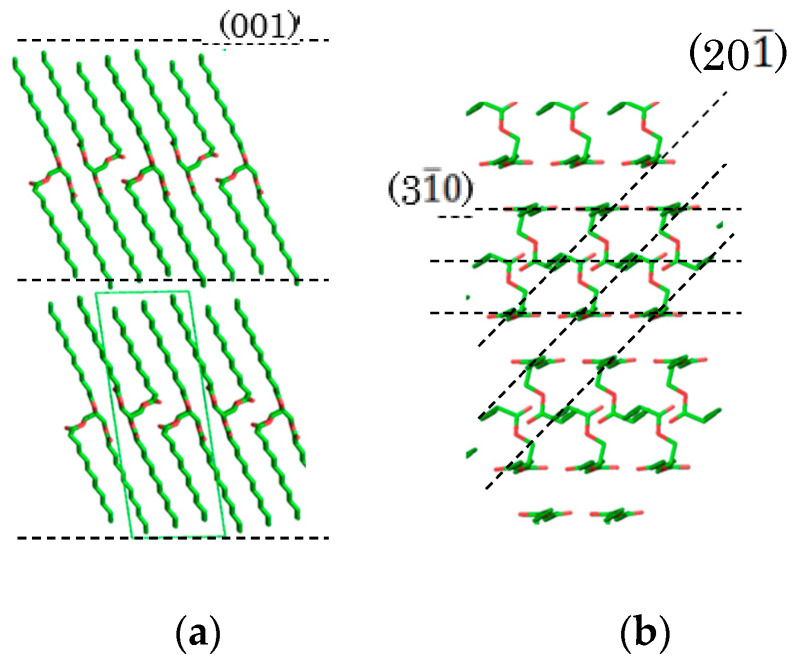
Crystal structure of trilaurin. (**a**) Lamella and (001) face and (**b**) subcell packing and (20-1) and (3-10) faces. The crystal structure was drawn using the *PyMol* software package [24].

The polymorphic transformation of saturated monoacid TAGs has been well studied [9,10,11,12]. Saturated monoacid TAGs can be crystallized as β, β’, or α polymorphic forms. The unstable forms, α and β’, easily transform into the more stable polymorph, β’ or β. The existence of multiple β’ forms of saturated monoacid TAGs was also suggested [13,14,15]. J. W. Hagemann et al. claimed that there are at least two β’ forms: β’_2_ with a low melting point and β’_1_ with a high melting point, each of which has a different lateral packing arrangement of hydrocarbon chains [14]. S. Ueno et al. studied the morphological and orientation changes of trilaurin (LLL) spherulites undergoing a polymorphic transformation. They found that morphology of the LLL crystal was changed but the orientation of LLL β’ form was conserved during transformation into the β form [16]. Kellens et al. suggested that the experimental data are not sufficient to prove the existence of multiple β’ or β forms, and the difference between both subforms seems to be rather a question of crystal perfection and crystallinity effects [10]. In contrast, four polymorphic forms of LLL with 4% of cholesterol were reported by C. Allains et al. [17]. They found two β forms in addition to the typical α and β’ forms. Synchrotron X-ray diffraction revealed that signature peaks of β form at 1.63 and 1.685 Å^−1^ shifted to 1.615 and 1.66 Å^−1^. However, clear evidence of more polymorphs of pure saturated monoacid TAGs have not been found. Few TAG crystal structures have been determined from single crystals since they do not produce large thick crystals [18]. Saturated monoacid TAGs such as tricaprin, LLL, and tripalmitin (PPP) produce exceptionally large β form crystals, and their structures have been determined (Figure 1) [19,20,21,22,23]. Their crystal structures were quite similar [23] even though they have fatty acid moieties with different chain lengths. The crystal structures of the α and β’ forms of saturated monoacid TAGs have not been determined as yet.

In the present study, we determined the crystallization and polymorphic transformation conditions required to make multiple β forms of saturated monoacid TAGs with different fatty acid chain lengths (LLL, trimyristin (MMM), PPP, and tristearin (SSS)). The morphological and physical properties of the multiple β forms have been determined using polarized light microscopy (PLM), XRD, and DSC.

## 2. Results and Discussion

### 2.1. Melting Point and Morphology of Multiple β Forms of LLL

**Figure 2:**
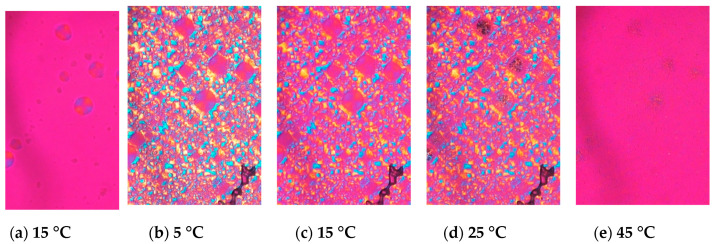
Morphological changes and melting behavior of trilaurin observed by polarized light microscopy at (**a**) 15 and (**b**) 5 °C while cooling and (**c**) 15, (**d**) 25, and (**e**) 45 °C while heating. (**a**) Trilaurin was crystallized in β’ form at 15 °C, then (**b**) α form covered them. The scale bar is 100 μm.

When LLL was cooled, LLL was first crystallized into β’ form spherulites (Figure 2a), and then the α form also crystallized, which covered the β’ (Figure 2b). Both the α and β’ forms of LLL crystallized as spherulites as shown in Figure 2a and b, but the colors seen with the sensitive color plate were different. The α form showed as bright yellow and sky-blue depending on the orientation of the crystals, while β’ form had darker blue and orange tones. The difference in birefringence of these polymorphs based on their crystal structures should be the cause of the differences in color. The brighter α form should be optically anisotropic in glycerol moiety, while β’ would have symmetric packing. When the temperature was increased, the α form kept its spherulite morphology and changed color, while the β’ form lost its spherulite morphology and turned into bright small crystals (Figure 2d). The melting points of these crystals were much higher than either the α or β’ forms and close to the melting point of the β form. Therefore, the color and morphological changes accompanied the polymorphic transformation as the temperature increased. Dark area corresponding to air pockets appeared in the microscope images during heating implying a volume shrinkage as the TAG transformed into a more tightly packed stable form. The crystals transformed from the α form melted at a lower temperature than the crystals that originated in the β’ form (Figure 2e). These results imply that crystals with a different transformation history could have different melting points.

**Figure 3:**
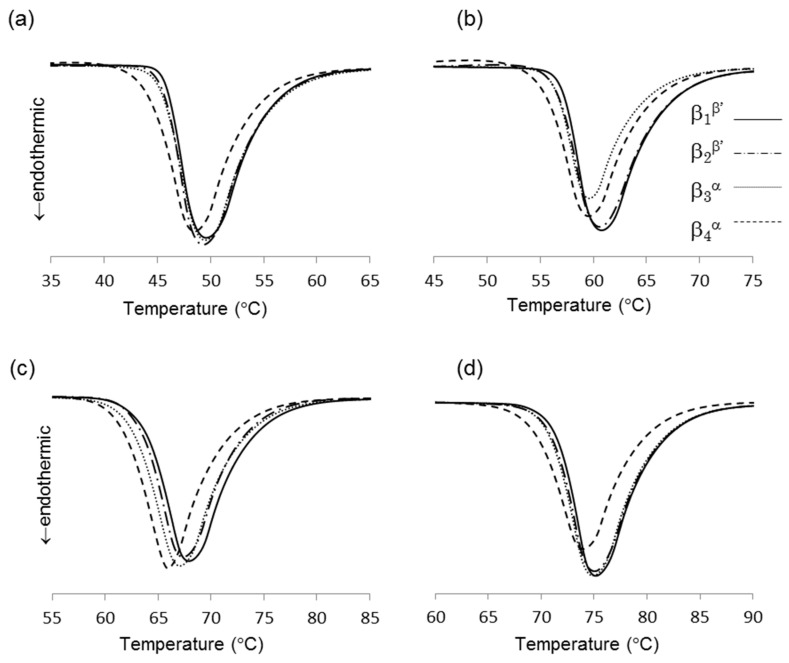
Thermograms of four β forms of saturated monoacid triacylglycerols (TAGs), (**a**) trilaurin, (**b**) trimyristin, (**c**) tripalmitin, and (**d**) tristearin.

**Figure 4:**
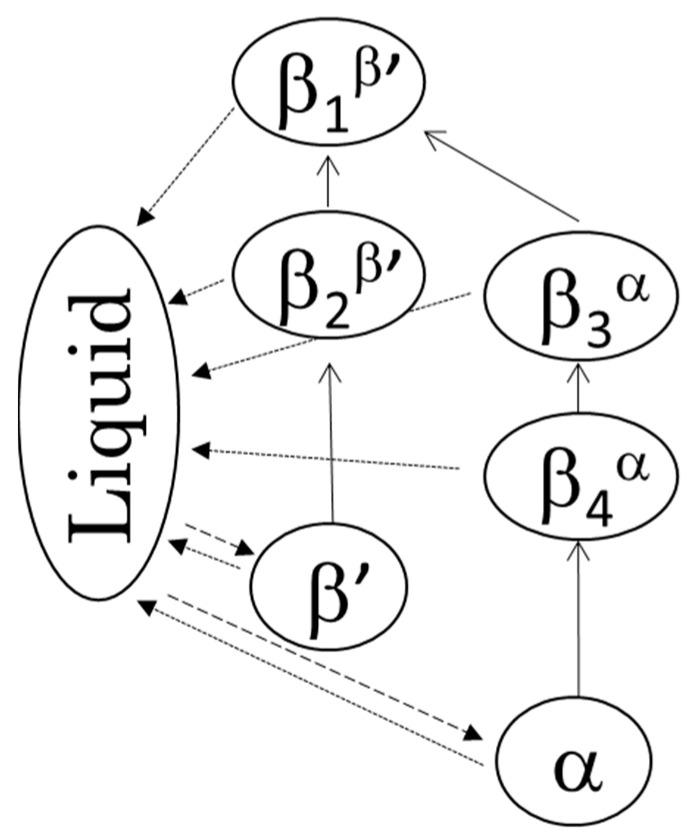
The two polymorphic transformation pathways and four β forms: crystallization (broken lines), transformation (solid lines), and melting (dotted lines).

DSC thermograms of the four β forms obtained from LLL (see 3.2 for sample preparation) are shown in Figure 3a, and their melting points are given in Table 1. Depending on the crystallization routes, the four β forms were named β_1_^β′^, β_2_^β′^, β_3_^α^, and β_4_^α^ in the order of their melting points for convenience. The onset temperatures of these β forms showed small differences as shown in Table 1. The β forms obtained through the α route showed lower melting points than the those made via the β’ route. This is consistent with the PLM results. In both routes, the melting points of initial β forms (β_2_^β′^, β_4_^α^) were increased by heat treatment (β_1_^β′^, β_3_^α^). These results imply that each β form has their own specific melting points. The polymorphic transformation in LLL is schematically drawn in Figure 4. However, the melting point of a crystal can be decreased from that of a pure material if the crystal has a significantly small size. Thus, the melting points and morphological changes cannot be conclusive evidence of the existence of multiple β forms, as discussed in previous works [9,10].

### 2.2. Synchrotron Radiation X-ray Diffraction (SR-XRD) in the β’ Route

**Figure 5:**
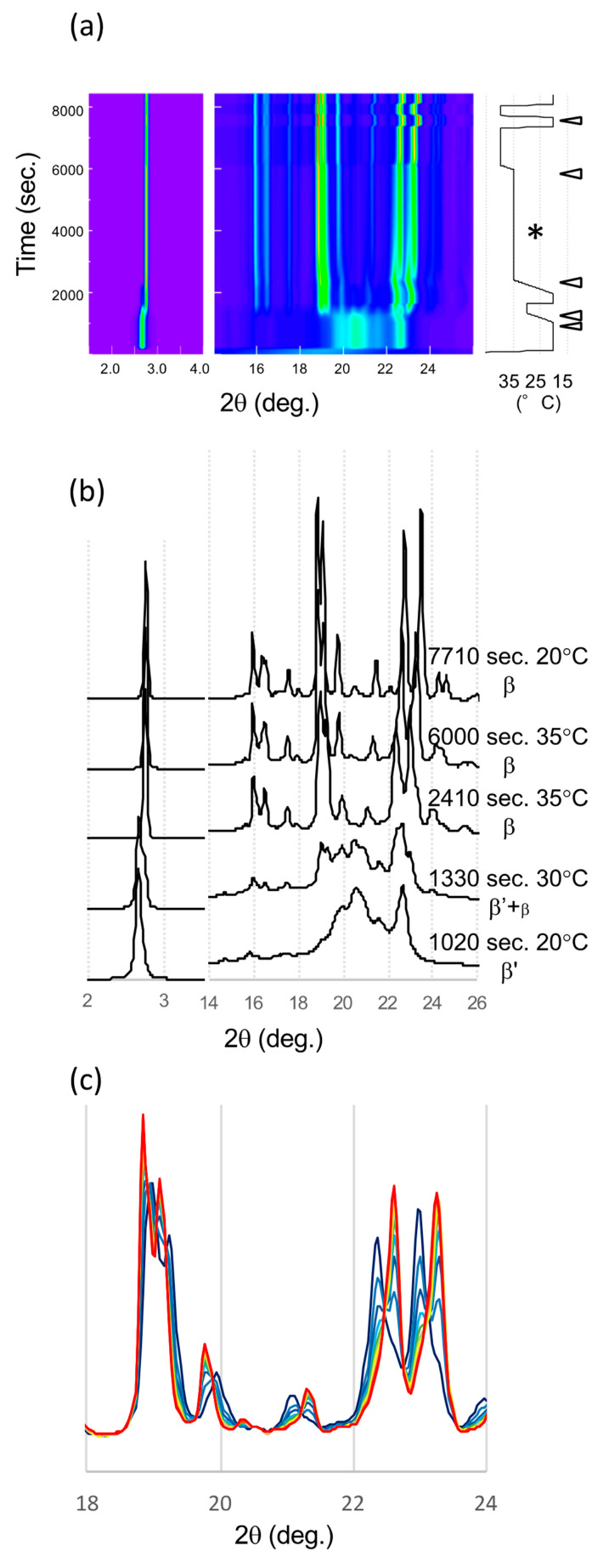
SR-XRD patterns from LLL crystals in β’ route. (**a**) Time evolution on small- and wide-angles X-ray scattering (SAXS and WAXS). The temperature change during the measurement is on the right. Diffraction intensity is shown in rainbow colors from violet to red. (**b**) SAXS and WAXS profile changes from different polymorphs. The triangles in (**a**) indicate the time of each profile. (**c**) WAXS pattern development during isothermal heat treatment at 35 °C for 1 h (*). The XRD profile was extracted every 5 min from violet to red.

The series of SR-XRD patterns taken in the β’ route is shown in Figure 5. Because these patterns were taken sequentially in the same setup at SR; fixed detector, sample holder, and no replacement of samples during the measurement, the positional reproducibility is high enough to compare the diffraction patterns. First, LLL crystallized in the β’ form at 20 °C. A single peak corresponding to the lamella distance of the β’ form was confirmed by small angle X-ray scattering (SAXS) (2.67°), and a typical broad peak at 20.6° and a sharp peak at 22.7° were found by wide angle X-ray scattering (WAXS) after incubation at 20 °C for 15 min (Figure 5a,b). During heating to 30 °C, the SAXS peak shifted to a higher angle corresponding to the β form (2.75°) (Figure 5a). In addition, the WAXS pattern also started to change from that of the β’ phase to that of the β form (β_2_^β’^). At 35 °C, the XRD pattern clearly indicated the transformation into the β form. The two signature peaks at 22.37 and 22.98° were well-developed. The time evolution with further heat treatment at 35 °C clearly demonstrates a decline in these signature peaks (Figure 5c). In addition, two new peaks at 22.61 and 23.26° grew beside the declining peaks. The further heat treatment at 40 °C finished the transformation. Then, the XRD patterns from initial and final β crystals were similar but the peak positions had shifted. Even the position of these peaks moved, the corresponding peaks of β_2_^β’^ were found in β_1_^β′^ (Figure 5c). This means that molecular packing of these two β forms were quite similar.

The structural and diffraction pattern similarity in saturated monoacid TAGs β forms was mentioned [23]. Thus, we can discuss the diffraction patterns of LLL β forms as an analogue of PPP β form, of which single crystal structure was determined and its structure factor is available. The diffraction intensity is proportional to the square of structure factor. Two signature peaks developed in LLL WAXS patterns, corresponding to 20-1 and 3-10 of PPP, respectively (Millar indexing was based on the literature [23]). The structure factor of 20-1 peak in PPP is more than 100 times higher than surrounding peaks. The structure factor of 3-10 (23.802°) in PPP is also significantly high compare to the surrounding peaks, except for -2-11 (23.807°), which has the comparable structure factor (5 times smaller than 3-10). The corresponding peaks calculated with unit cell of LLL are not overlapped (23.412° and 24.153°) and can be considered as independent peaks. The Bragg angle was calculated with λ = 0.15 nm. Therefore, these two peaks, 20-1 and 3-10 in LLL, can be considered as strong and isolated single peaks. Since the peak position depends on the temperature, we compared the peak positions of β_1_^β′^ and β_2_^β’^ at 20 °C (Table 2). The spatial resolution of the detector calculated from the pixel size under this condition was around 0.02°, and we integrated the XRD intensity approximately every 0.05°. Because the shift of these peaks in absolute value was more than 0.12°, these changes must be significant. This development in the SR-XRD pattern clearly indicates that the LLL crystals change their polymorphic form from one β phase (β_2_^β’^) to a different β form (β_1_^β’^) during the heat treatment. The size of the crystals can affect the broadness of the diffraction peaks but not the position of them. If the size of crystals increased during the heat treatment, the XRD pattern could sharpen, but peaks can never disappear. In addition, if the peak position could be affected by the quality or the size of crystals, the continuous peak shift should appear with the improvement of the quality and size because they would be improved continuously. Therefore, the declining and appearing of two isolated peaks, 20-1 and 3-10, indicate the polymorphic transformation of the crystal.

### 2.3. SR-XRD in the α Route

**Figure 6:**
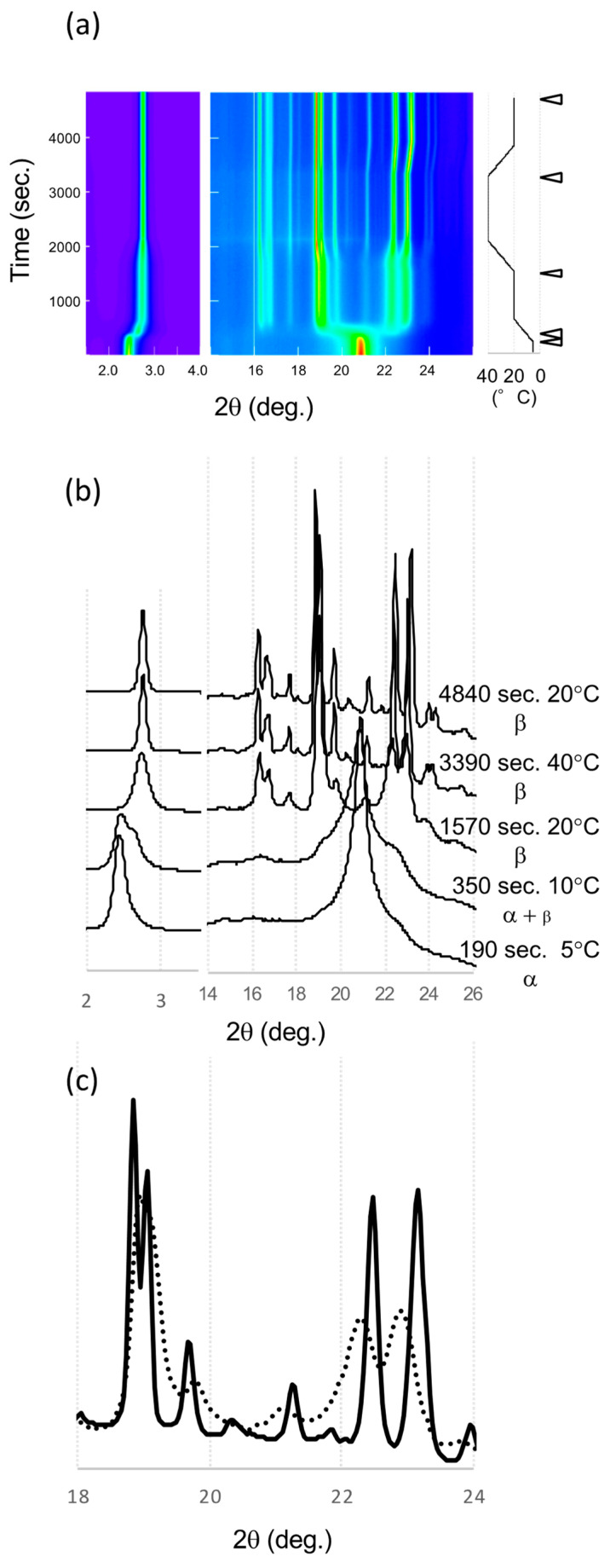
SR-XRD patterns from LLL crystals in α route. (**a**) Time evolution on small- and wide-angles X-ray scattering (SAXS and WAXS). The temperature change during the measurement is in the right. Diffraction intensity is shown in rainbow colors from violet to red. (**b**) SAXS and WAXS profile changes from different polymorphs. The triangles in (**a**) indicate the time of each profile. (**c**) Comparison of WAXS profiles of β_3_^α^ and β_4_^α^ at 20 °C. The solid and dotted lines are for β_3_^α^ and β_4_^α^, respectively.

SR-XRD patterns taken in the α route are shown in Figure 6. LLL was crystallized in the α form at 5 °C with single peaks at 2.45° in the SAXS and 20.92° in the WAXS data. During heating from 5 to 20 °C, a new peak at 2.66° appeared and shifted to 2.73° in the SAXS data. These peaks correspond to β’ and β, respectively; the WAXS pattern did not show the clear existence of β’. At 20 °C and 15 min, the WAXS peaks were broad but a clear pattern of β form (β_4_^α^) was observed. The WAXS pattern did not show the drastic change seen in the β’ route but the peaks shifted during the heat treatment at 40 °C.

Finally, the peaks sharpened, and peak positions were changed. The patterns before and after heat treatment showed the peak shift of signature peaks of the β forms. The peak width in the initial β form (β_4_^α^) was broad, which indicates a small size or low quality of these β crystals. After the heat treatment at 40 °C, the peaks were sharper and more intense, indicating that the crystallinity and size had improved. The polymorphic transformation to be β_3_^α^ with heat treatment had occurred because the XRD peak shift was similar to that found in the β’ route. The peaks at 22.25° and 22.94° shifted to higher angles, 22.46° and 23.16° at 20 °C, while the peaks around 19° moved to lower angles. These movements clearly indicate polymorphic transformations during heat treatment as shown in β’ route.

**Figure 7:**
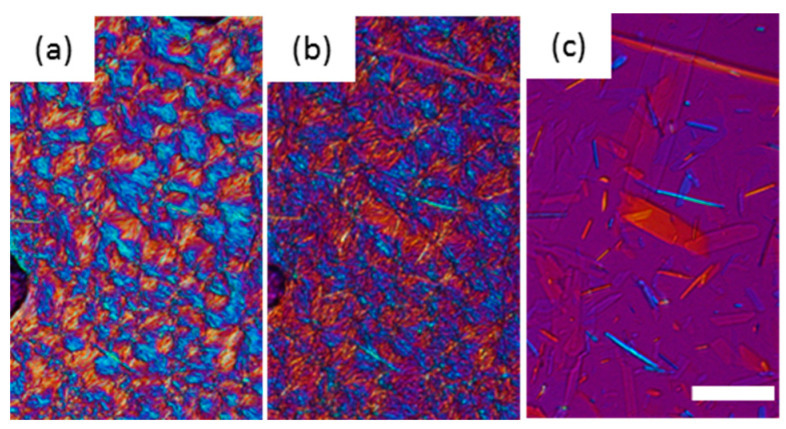
The morphological changes and melting behavior of β_3_^α^ after heat treatment at 44 °C for 12 h. (**a**) 44 °C, (**b**) 45.2 °C, and (**c**) 45.5 °C. The scale bar is 50 μm.

The melting points of these β forms made via the α route were lower than the β forms from the β’ route as shown in Figure 3. This means polymorph of these α route β forms (β_3_^α^ and β_4_^α^) were different from the β’ route β forms (β_1_^β′^ and β_2_^β’^), even these XRD patterns of the subcell were similar. To prove this hypothesis, we applied a further heat treatment at 44 °C for 15 h to the α route β forms (Figure 7). The appearance of β forms after the heat treatment was not changed; however, when the temperature was increased, the α route β forms melted and crystals with a needle shape appeared. These needle crystals had a higher melting point than the α route β forms, indicating a further polymorphic transformation had occurred with the heat treatment. If β_3_^α^ had the same polymorph as β_1_^β′^ and the increase in melting point was caused by the increase in crystal size, almost all the β_3_^α^ crystals should increase their melting point and remain after this heat treatment. The small number of needle shape crystals was transformed from β_3_^α^ to β_1_^β′^ during heat treatment. The melting point after 12 h treatment determined by DSC was 45.99 ± 0.09 °C. This value is identical to the melting point of β_1_^β′^ and significantly increased from that of the β_3_^α^ made via the α route (45.30 ± 0.10 °C). Therefore, we conclude that the α route β forms transformed to β forms from the β’ route.

### 2.4. Estimation of Crystal Size

The melting point of a crystal will be decreased if the crystal size is small. Considering this effect, we estimated the crystal size from SR-XRD pattern. We used the diffraction peaks, 20-1 and 3-10 of β_1_^β′^ and β_3_^α^ for this estimation. The β values of β_1_^β′^ were 3.4 × 10^−3^ and 3.6 × 10^−3^, and 3.3 × 10^−3^ and 3.9 × 10^−3^ for β_3_^α^, and the calculated crystal sizes from these β values were 41, 39, 42, and 35 nm. This is the minimum size of crystals since β value is the sum of the divergence of X-ray beam from crystals and the equipment. These sizes of crystals in β_1_^β′^ and β_3_^α^ were similar since the effect from the equipment on these measurements should be similar. Therefore, the difference in the melting point of β_1_^β’^ and β_3_^α^ (Table 1) was not caused by the size of the crystals. This also supports the polymorphism in α and β’ routes. Similar analysis was carried out to the diffractions 20-1 and 3-10 of β_2_^β’^ and β_4_^α^. The obtained size of the β_2_^β’^ crystal was around 29 nm while the sizes of β_4_^α^ were 13 and 17 nm. This small size of β_4_^α^ would be the reason for the relatively low melting point of β_4_^α^ crystal.

### 2.5. Parallel Pathways of Polymorphic Transformation in Saturated Monoacid TAGs

**Figure 8:**
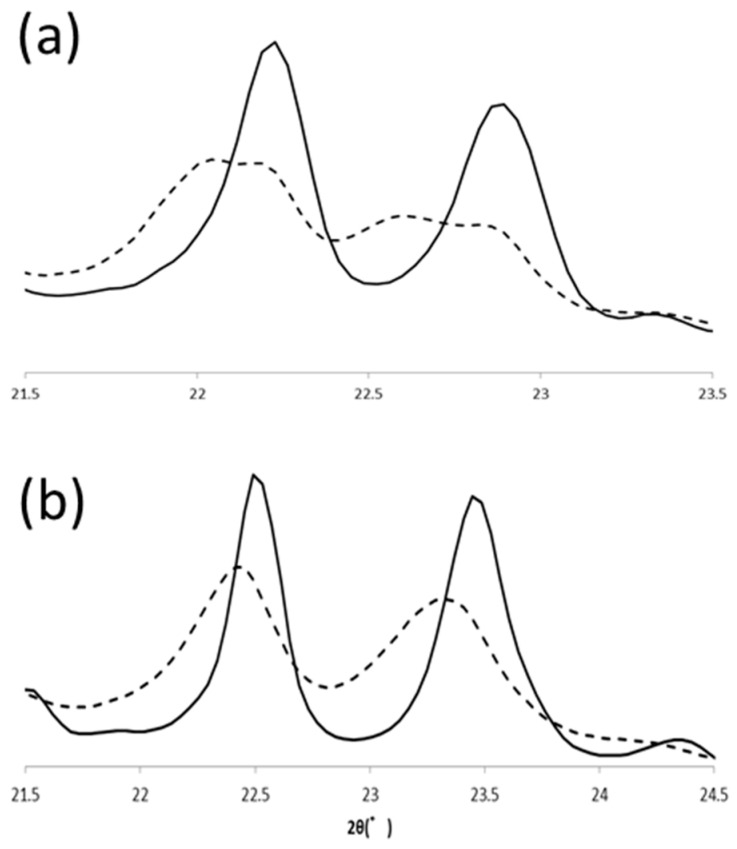
Comparison of initial (β_2_^β′^, β_4_^α^) and final (β_1_^β′^, β_3_^α^) β forms of PPP. SR-XRD patterns in (**a**) β’ and (**b**) α routes. The dotted and solid lines are for initial and final β forms, respectively.

Polymorphic transformation of PPP in α and β’ routes were also observed with SR-XRD. In the β’ route, β’ form PPP was transformed to β form (β_2_^β’^) during heating to 57 °C, and the β form changed its polymorph to another β form (β_1_^β′^) at 57 °C. Because the transformation was very fast under this condition, we could not isolate the initial β form clearly. From the beginning at 57 °C, two β forms coexisted but transformation was progressed to reach the final β form during heat treatment (Figure 8). PPP was crystallized in α form at 30 °C and then it transformed to β form (β_4_^α^) during the incubation at 43 °C. This β form was transformed into another β form (β_3_^α^) during further heat treatment at 57 °C. The signature peaks, 20-1 and 3-10, shifted as shown in LLL β forms. The initial positions of these peaks at 20 °C were 22.45° and 23.33° and the final positions were 22.49° and 23.45°. The peak tops of 20-1 were close, but the peak profiles of the initial and final peaks were clearly different. The peak position of 3-10 was significantly shifted by the heat treatment. Therefore, PPP also has four β forms and shows the same polymorphism as LLL.

Based on the crystallization condition of LLL and PPP, we modified the crystallization conditions of other saturated monoacid TAG to obtain multiple β forms. Figure 3 showed the difference of melting points in multiple β forms of saturated monoacid TAGs. The cooling rate and crystallization temperature were controlled, and the α and β′ forms were independently crystallized. The obtained melting points of these TAGs clearly showed the significant difference in initial and final β forms. As found in LLL and PPP, MMM and SSS also showed four melting points for β forms. The melting points of initial β form (β_2_^β’^ and β_4_^α^) could be increased by heat treatment. Thus, saturated monoacid TAGs will show the same polymorphism as shown in Figure 4.

## 3. Materials and Methods

### 3.1. Materials

TAGs (LLL, MMM, PPP, and SSS) (>99% purity) were purchased from Sigma-Aldrich Co. (St. Louis, MO, USA) and used without further purification. TAGs were stored in a freezer and brought back to room temperature before use.

### 3.2. Crystallization and Heat Treatment Conditions

**Figure 9:**
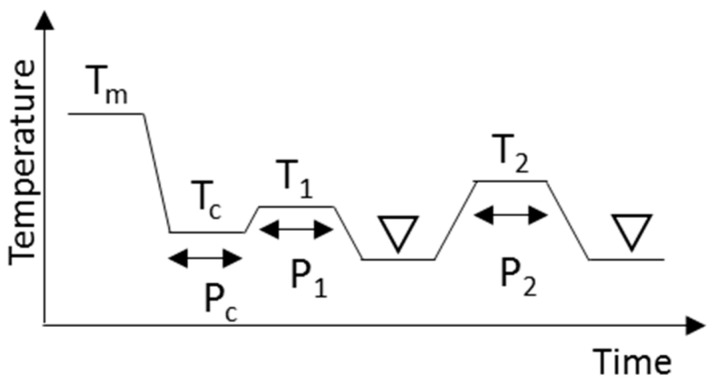
Schematic drawing of the crystallization and heat treatment conditions needed to obtain β forms. Triangles indicate the regions where XRD and DSC measurements were made at 20 °C. Tm, Tc, T_1_, and T_2_ are temperatures and Pc, P_1_, and P_2_ are time periods.

All the TAGs were crystallized in α or β’ forms prior to investigating the phase transition to the β form. Here we refer to these processes as α or β’ routes, respectively. To obtain α form crystals for use in the α route, a TAG was quickly cooled to a low enough temperature and kept for a time until crystallized completely. After that, the temperature was increased gradually and kept below the melting point of the α form until the α form transformed completely into the β form. The β form obtained was characterized by DSC and XRD. The β form was next heated and kept at a temperature below its melting point for the further transformations to different β form. For the β’ route, TAGs were kept at temperatures below their melting points. The β’ form obtained were kept at the same or a little higher than the crystallization temperature to promote the polymorphic transformation into the β form. The transformation was confirmed by DSC and XRD. The heat treatment was applied again for further transformations to a different β form. The obtained β form was then kept below its melting points. In these processes, all TAGs were kept below the melting points of each solid phase to prevent melting, and the transformation was done entirely through a solid–solid transformation. The end of all the solid–solid transformations were confirmed with DSC and XRD. We tried many conditions to obtain the best results: the heating and cooling rates and the periods and temperatures for the heat treatments are summarized in Figure 9 and Table 3.

### 3.3. Polarized Light Microscopy

The crystallization and phase transition behavior of LLL was observed under a polarized light microscope (PLM) (BX51 or IX73, Olympus, Tokyo, Japan) with a temperature control stage, LINKAM (LK-600PM and 10021, Linkam Scientific Instruments, Ltd., Tadworth, UK). A sensitive color plate was used with the PLM to determine the morphology and the orientation of crystals. LLL was sandwiched between two cover slips. The conditions of crystallization and heat treatments were simplified in order to see the polymorphic transformation from α or β’ to β forms. Molten LLL was cooled to 5 °C at 2 °C min^−1^ and held for several min to obtain both α and β’ forms, respectively. Then they were heated at 2 °C min^−1^ until they melted. The sample chamber of the LINKAM was filled with N_2_ gas to prevent water condensation on the surface of the sample.

### 3.4. Differential Scanning Calorimetry (DSC)

The melting behaviors of saturated monoacid TAG β forms were investigated using DSC (DSC7000X, Hitachi High-Tech Science Corporation, Tokyo, Japan). TAGs (approximately 3 mg) were sealed in an aluminum pan. The heat treatment was applied to the pan on the LINKAM to obtain the β form (Table 3). The melting points of the TAG β forms were determined by heating from 0 °C to 100 °C at a rate of 20 °C min^−1^. The fast heating rate was selected to prevent a phase transition during heating. The onset temperatures were adopted as melting points, which were determined as the intersection of baseline and tangent line at the inflection point of the endothermic peak. The sampling was every 10 ms, and an empty pan used as a reference. We obtained melting points to 2 decimal places. Temperature was calibrated by the use of indium and tin samples. Three replicates were conducted for each sample. The sample standard deviation was shown as an error.

### 3.5. Synchrotron Radiation X-ray Diffraction (SR-XRD)

SR-XRD measurement was carried out at the beamline 6A of the synchrotron radiation facility (Photon Factory) of the High Energy Accelerator Research Organization (KEK) in Tsukuba, Japan. The X-ray wavelength was 0.15 nm, and SAXS and WAXS patterns were measured simultaneously using detectors PILATUS 1M and PILATUS 100K (Pilatus Aircraft Ltd., Stans, Switzerland), respectively. The calibration of the detector distances was performed using silver behenate for SAXS and PPP for WAXS. SAXS and WAXS patterns were taken every 10 s for a 5 s exposure and at 5 s intervals. The spatial resolution of the detector for WAXS calculated from the pixel size under the condition was around 0.02°, and we integrated the XRD intensity approximately every 0.05°. A circular iron sample holder, 0.5 mm thickness and a 5 mm diameter window, was used. TAGs were injected into the holder and the window was sealed with Kapton film. The temperatures of the samples were controlled by a LINKAM. For in situ SR-XRD measurements of the transformation of LLL and PPP from the β’ to β form, the conditions (Table 3) were slightly modified to obtain the clear solid–solid transformation. The molten LLL was cooled to 20 °C to obtain the β’ form. The obtained β’ form was heated from 20 °C to 30 °C and kept there for 5 min. The sample was next cooled to 20 °C and then heated to 35 °C for 1 h. A further heat treatment at 40 °C for 20 min was applied. Finally, the β form obtained was cooled to 20 °C. Another heat treatment at 40 °C for 5 min was repeated to confirm the end of the polymorphic transformation. The rate of heating in these processes was 2 °C min^−1^ and the cooling rate was 100 °C min^−1^ except for the last heating used to confirm the end of reaction.

### 3.6. Scherrer Method

Scherrer method was used to determine the grain size of crystals [25]. Scherrer’s Equation (1) is classical but widely used to estimate the crystal size from XRD pattern. The Equation (1) is:

(1) $$D=\frac{K\lambda }{\beta sin\theta }$$

where *D* is the size of the crystal, *K* is the shape factor, *λ* is the wavelength, *β* is the full width at half maximum (FWHM) of the peak in radian, and *θ* is the Bragg angle. We applied *K* = 0.9 in this analysis. The diffraction angle and FWHM of these peaks were determined using curve fitting software (*Fityk ver. 1.3.1*) [26]. A Pearson 7 was used as the fitting function.

### 3.7. Statistical Analysis

Melting point measurements were carried out at least three times, and the differences between samples were evaluated by TukeyHSD with *p* < 0.05 (*R ver. 4.0.2*) [27].

## 4. Conclusions

This study discusses the existence of multiple β forms in saturated monoacid TAGs, LLL, MMM, PPP, and SSS from XRD, DSC, and optical microscopy data for the first time. The particular forms depend on the treatment and crystallization history: starting from the β’ form, we obtained the β_1_^β′^ and β_2_^β’^ forms, while starting from the α form, we obtained the β_3_^α^ and β_4_^α^ forms. The first stage of the α and β’ routes produced transformations into the different β forms, β_2_^β’^ and β^α^. Additional heat treatments promoted the further polymorphic transformations into β_1_^β′^ and β_3_^α^. The structural similarity found between β_1_^β′^ and β_3_^α^ and β_2_^β’^ and β_4_^α^ indicate the existence of two stable packings of the fatty acid moiety in β forms of saturated monoacid TAGs found in different TAGs like cocoa butter. This study is expected to be beneficial for the understanding of polymorphic transformations in TAG crystal and the improvement of fat-based product stability.

## Figures and Tables

**Table 1:** Melting points (°C) of the multiple β forms of TAG.

	β_1_^β′^	β_2_^β′^	β_3_^β′^	β_4_^β′^
LLL	45.82 ± 0.08^a^	45.63 ± 0.13 ^a,b^	45.30 ± 0.10^b^	44.39 ± 0.2^c^
MMM	57.07 ± 0.05^a^	56.51 ± 0.10^b^	56.51 ± 0.14^b^	55.30 ± 0.03^c^
PPP	63.53 ± 0.07^a^	63.14 ± 0.02^b^	62.50 ± 0.13^c^	61.57 ± 0.19^d^
SSS	71.38 ± 0.11^a^	70.93 ± 0.03^b^	70.70 ± 0.13^b^	69.35 ± 0.17^c^

Different letters in the same row show significant difference (*p* < 0.05). Errors represent the sample standard deviations.

**Table 2:** Peak center (2θ in degree) of LLL β_1_^β′^ and β_2_^β′^ peaks, and their shift.

β_1_^β’^	β_2_^β’^	δθ
18.847	18.974	−0.127
19.059	19.228	−0.169
19.733	19.943	−0.210
21.476	21.229	0.247
22.736	22.494	0.242
23.457	23.177	0.280
24.288	24.052	0.236
24.602	24.328	0.274

**Table 3:** Heat treatment conditions for producing multiple β forms of TAG.

	Route	T_m_	Rate (°C min^−1^)	T_c_, P_c_	Rate (°C min^−1^)	T_1_, P_1_	Rate (°C min^−1^)	T_2_, P_2_
LLL	α route	60 °C	100	5 °C, 5 min	2	20 °C, 15 min	2	40 °C, 20 min
	β’ route		100	20 °C, 15 min	2	30 °C, 5 min	2	40 °C, 20 min
MMM	α route	70 °C	100	20 °C, 5 min	100	30 °C, 20 min	100	52 °C, 30 min
	β’ route		100	33 °C, 5 min	100	35 °C, 35 min	100	52 °C, 30 min
PPP	α route	80 °C	100	30 °C, 5 min	100	43 °C, 70 min	100	57 °C, 30 min
	β’ route		100	46 °C, 30 min	100	46 °C, 120 min	100	57 °C, 30 min
SSS	α route	85 °C	100	40 °C, 5 min	100	54 °C, 240 min	100	67 °C, 30 min
	β’ route		100	56 °C, 60 min	100	56 °C, 120 min	100	67 °C, 30 min
